# Factors associated with unfavorable tuberculosis treatment outcomes in the population deprived of liberty

**DOI:** 10.5588/ijtldopen.25.0210

**Published:** 2025-10-10

**Authors:** K.Z. Ely, M.L. Draschler, T.N. Prado, V.G. Vendrusculo, I. Frighetto, C.A. Jarczewski, R.M. Dotta, M.M. Dall’Soto, E.G. Boeira, C. Busatto, A.R.M. Valim, L.G. Possuelo

**Affiliations:** ^1^ Secretaria Estadual de Saúde do Rio Grande do Sul, SES/RS, Porto Alegre, Brazil;; ^2^ Universidade de Santa Cruz do Sul (UNISC), Santa Cruz do Sul, Brazil;; ^3^ Universidade Federal do Espírito Santo (UFES), Vitória, Brazil;; ^4^ Coordenação-Geral de Vigilância da Tuberculose, Micoses Endêmicas e Micobactérias não Tuberculosas, Ministério da Saúde, Brasília, Brazil.

**Keywords:** tuberculosis, communicable diseases, health policy, prisons, vulnerable populations

## Abstract

**BACKGROUND:**

TB represents a significant challenge within the prison context. The objective of this study was to identify factors associated with the unfavourable outcomes (no-cure) of TB treatment in the population deprived of liberty (PDL) in southern Brazil.

**METHODS:**

A cross-sectional study using secondary data from the national notification system was developed to identify TB outcomes. Bivariate and multivariate logistic regressions were applied to determine the dimensions of care associated with unfavourable outcome of TB treatment in the PDL. Adjusted odds ratios and 95% confidence intervals were provided for each dimension evaluated.

**RESULTS:**

A total of 3,022 TB cases in the PDL were analysed, of which 1,077 (35.6%) resulted in unfavourable outcome. After adjustment, the following were associated with unfavourable outcome: living with HIV/AIDS, notification by other teams, entry by transfers, clinical epidemiological diagnosis, and self-administered treatment.

**CONCLUSION:**

The factors associated with unfavourable TB treatment outcomes in PDL are mainly related to non-compliance with public policy determinations and government strategies, such as: lack of health staff in prison institutions, excessive transfers, lack of confirmatory TB tests, and failure to perform directly observed treatment, which should be the rule.

TB remains a major challenge for global public health. In 2023, approximately 10.8 million people fell ill, and 1.25 million died from the disease worldwide.^1^ TB is increasingly concentrated in population deprived of liberty (PDL), which account for 11% of the total TB cases in Central and South America, while this population represents 1% of the total population.^2^ Incarceration is the primary risk factor for TB in the Americas region,^3^ and in South America, the incidence rate is 26 times higher than that in the general population.^4^ PDL are the most affected by TB in Brazil. In 2023, 7,240 new TB cases were reported in penal institutions, representing 8.9% of all new cases.^5,6^ Between 2020 and 2022, there was a decrease in the cure rate of new cases (from 71.7% to 64.8%), an increase in TB-related deaths (from 2.0% to 2.2%), and an increase in cases closed due to transfers (from 14.5% to 25.1%).^5^ Resistant forms of the disease, irregular treatment, and late detection are commonly encountered problems and represent additional challenges to the cure of the disease within PDL.^6^

Strategic actions are crucial for achieving the Sustainable Development Goals and the targets of the 2030 Agenda. The elimination of TB as a public health problem is part of target 3.3 of Goal 3, Health and Well-being, with the challenge of reducing the mortality rate by 90% and the incidence rate by 80% by 2030, aiming to eliminate the disease by 2050.^7^ In light of the high incidence and the need to control the disease, the objective of this study was to identify the factors associated with the unfavourable outcome of TB treatment in PDL in southern Brazil.

## METHODS

**Figure 1.**
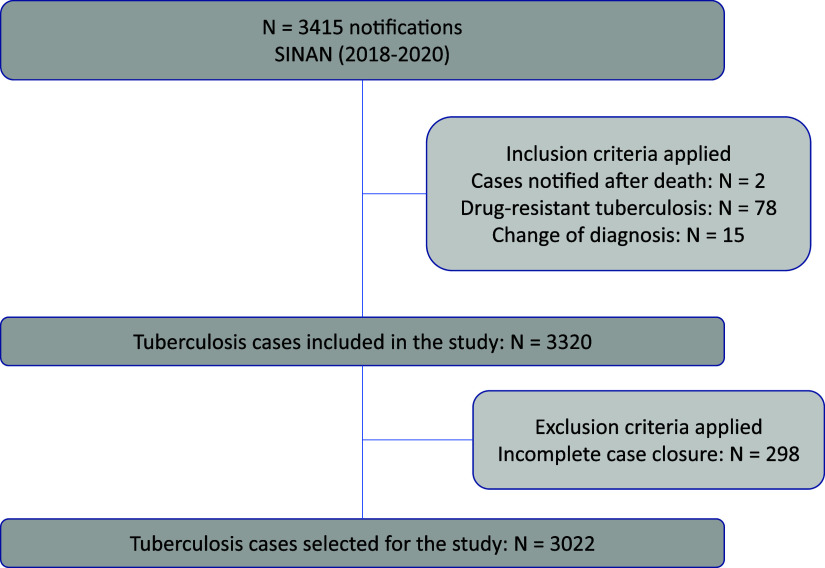
Cases of sensitive TB in population deprived of liberty in Rio Grande do Sul.

**Figure 2.**
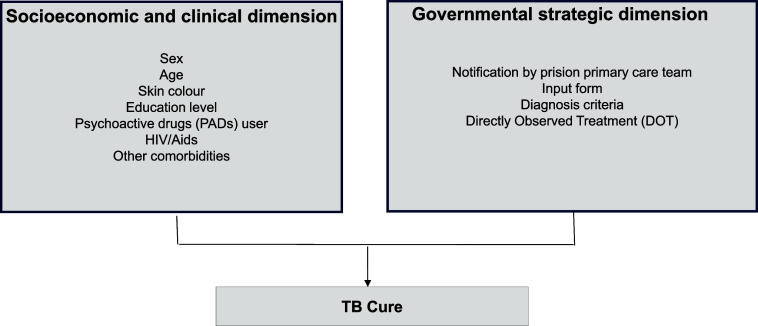
Dimensions of care for TB in the prison system.

A cross-sectional study design was used, based on secondary data from mandatory TB notification and follow-up in the state of Rio Grande do Sul, recorded in the National System of Disease Notification (Portuguese acronym: SINAN, *Sistema Nacional de Agravos de Notificação*), Ministry of Health of Brazil. The population of interest includes the PDL with TB who were prescribed the standard treatment regimen (2RIPE/4RI). All TB notifications in PDL recorded in the SINAN from 2018 to 2020 were selected, provided the diagnosis was made in life and the case closure did not indicate the occurrence of multidrug-resistant TB (MDR-TB). MDR-TB cases are monitored in another information system, which is not SINAN. Notifications with incomplete case closures were excluded (Figure 1). Data collection was conducted in April 2022 by the State Health Surveillance Centre of Rio Grande do Sul (CEVS/RS). After assessing the data quality and removing sensitive information, the anonymised database was made available to the researchers. The dependent variable was the treatment outcome, divided into two groups: 1) cure and 2) unfavourable outcome. TB cases included in the unfavourable outcome group are those that did not progress to cure: treatment abandonment, transfer out, treatment failure, and primary abandonment. The cases that leave the SINAN system by transfer and do not enter another health service are considered cases with loss of follow-up. If the patient enters another health service, the system replaces the first record, considering the entry by transfer. The independent variables were grouped into two dimensions of health care for the PDL diagnosed with TB for disease control in the prison system: the socio-economic and clinical dimension and the government strategy dimension (Figure 2).

The socio-economic and clinical dimension was assessed considering sex, age, skin colour, educational level, use of psychoactive substances (PAS), living with HIV/AIDS, and other comorbidities. The categorisation followed these parameters: sex (male and female); age (18–29 years, 30–39 years, 40–59 years, and 60 years or older); skin colour (white and non-white); educational level (up to 8 years of schooling and 8 years or more); PAS use (yes and no); living with HIV or AIDS (yes and no); and comorbidities, as defined by the SINAN, for diabetes, mental illness, or other associated diseases and conditions (yes and no).

The government strategy dimension, the main focus of this study, includes the following variables: notification by the Primary Prison Care Team (Portuguese acronym: eAPP, *equipe de Atenção Primária Prisional*) – obtained from the National Health Establishments Registry (Portuguese acronym: CNES, *Cadastro Nacional de Estabelecimentos de Saúde*) using the number identified in the SINAN, entry type, diagnostic criteria, and directly observed treatment (DOT). The notification by the eAPP was considered as it represents the first point of care for PDL. The evaluated notifications were categorized into: type of reporting unit, according to CNES (eAPP and other reporting services, such as Health Surveillance, Family Strategy – FHS, Basic Health Unit – portuguese acronym: UBS, Unidade Básica de Saúde, Specialised Care Service, TB Reference Centers, Non-Hospital Emergency and Urgency Services, and Hospitals), notification status (new case and other types of entry, recorded in the SINAN database as relapse, re-entry after abandonment, or transfer); diagnostic criteria (bacteriological including: positive sputum smear microscopy, positive sputum culture, detectable rifampicin-sensitive Rapid Molecular Test [Xpert MTB/RIF]); X-ray and histopathology (histopathology with positive acid-fast bacilli results and suggestive of TB and suspicious chest X-ray); clinical epidemiological for cases without positive or suggestive diagnostic tests for TB; and DOT (yes and no).

The effect of the variables from both dimensions was analysed using multivariate logistic regression. Socio-economic and clinical variables were included in the statistical analysis, considering all of them as confounding variables for the effect of government strategies on cure. When a variable had more than 1% missing data, a category called ‘ignored’ was created to avoid case loss. The Statistical Package for the Social Sciences (SPSS), version 23.0 (IBM, Armonk, NY, USA), was used in data analysis. Initially, all variables were tested for the outcome of cure and non-cure using unadjusted analysis with a *P* value < 0.05 considered statistically significant. Multivariate modelling was used to evaluate the relationship between independent variables and the outcome, considering 95% confidence intervals (CIs) and including all variables, based on the theoretical model. Comparison categories (baseline) were defined by the number of individuals or worst prognosis: sex – male; age – 18–29 years; skin colour – white; educational level – up to 8 years of schooling; HIV/AIDS – yes; PAS use – yes; comorbidities – yes; notification by the eAPP – no; entry type – transfers; diagnostic criterion – clinical epidemiology; DOT – no.

### Ethical statement

The study adhered to the requirements of Resolution No. 466/2012 of the National Health Council and received approval from the Ethics Committees of the University of Santa Cruz do Sul (Opinion 4.251.858) and the School of Public Health of Rio Grande do Sul (Opinion 4.498.390).

## RESULTS

A total of 3,022 cases were considered valid for analysis, and the unfavourable outcome was observed in 1,077 (35.6%) of these cases. In the socio-economic and clinical dimension (Figure 2), most were male, age group of 18–29 years, white skin colour, and up to 8 years of schooling. Regarding health conditions, 14.3% were living with HIV/AIDS, 47% were users of PAS, and 12.2% had other associated diseases or conditions (Table 1). The eAPP was the notifying source of 1,413 (46.8%) cases. The remaining cases were notified as follows: 439 (14.5%) in hospitals, 415 (13.7%) in specialised services, 265 (8.8%) in UBS, 232 (7.2%) in TB reference services, 184 (6.1%) in administrative services, 42 (1.4%) in emergency and urgent care services, and 32 (1.1%) in the FHS. Pulmonary TB, new cases entering the system, bacteriological diagnoses, and self-administered treatment predominated (Table 2).

**Table 1.** Socio-economic and clinical dimension associated with the outcome of TB in the prison population reported in the Notifiable Diseases System, Rio Grande do Sul, 2018–2020.

Socio-economic and clinical dimension	Cure, n (%)	Unfavourable outcome, n (%)	Total, n (%)	*P* value
Sex (n = 3,022)				0.005
Male	1,895 (64.8)	1,029 (35.2)	2,924 (96.8)	
Female	50 (51.0)	48 (49.0)	98 (3.2)	
Age (n = 3,022)				0.003
18–29 years	1,018 (67.6)	488 (32.4)	1,506 (49.8)	
30–39 years	623 (61.7)	386 (38.3)	1,009 (33.4)	
40–59 years	278 (59.8)	187 (40.2)	465 (15.4)	
60 years or older	26 (61.9)	16 (38.1)	42 (1.4)	
Skin colour (n = 2,809)				0.433
White	1,198 (64.3)	664 (35.7)	1,862 (61.3)	
Non-white	618 (65.3)	329 (34.7)	947 (31.7)	
Level of education (n = 1,993)				0.160
Up to 8 years of schooling	919 (65.5)	484 (34.5)	1,403 (70.4)	
8 years or more	367 (62.5)	223 (37.8)	590 (29.6)	
HIV/AIDS (n = 2,844)				<0.001
Yes	203 (50.0)	203 (50.0)	406 (14.3)	
No	1,632 (66.9)	806 (33.1)	2,438 (85.7)	
Use of PAS (n = 2,640)				0.225
Yes	776 (62.6)	464 (37.4)	1,240 (47.0)	
No	908 (64.9)	492 (35.1)	1,400 (53.0)	
Comorbidities (n = 2,777)				0.054
Yes	201 (59.5)	137 (40.5)	338 (12.2)	
No	1,581 (64.8)	858 (35.2)	2,439 (87.2)	

Data described in absolute (n) and relative (%) frequencies. The total number of cases evaluated was 3,022, and for each variable analysed, cases labelled as ‘blank’ and ‘ignored’ were excluded. Significant values for *P* < 0.05 (χ² test).

PAS = psychoactive substances.

**Table 2.** Government strategies associated with the outcome of TB in the population reported in the Notifiable Diseases System, Rio Grande do Sul, 2018–2020.

Government strategies dimension	Cure, n (%)	Non-cure, n (%)	Total, n (%)	*P* value
Notification by the eAPP (n = 3,022)				<0.001
Yes	998 (70.6)	415 (29.4)	1,413 (46.8)	
No	947 (58.9)	662 (41.1)	1,609 (53.2)	
Entry type (n = 3,016)				<0.001
New cases	1,339 (67.7)	639 (32.3)	1,978 (65.6)	
Relapse	311 (63.9)	176 (36.1)	487 (16.2)	
Re-entry after abandonment	162 (55.7)	129 (44.3)	291 (9.6)	
Transfers	131 (50.4)	129 (49.6)	260 (8.6)	
Diagnostic criteria (n = 3,022)				0.002
Bacteriological	1,629 (65.6)	854 (34.4)	2,483 (82.2)	
X-ray and histopathology	260 (60.61)	169 (39.3)	429 (14.2)	
Clinical epidemiology	56 (50.91)	54 (49.09)	110 (3.6)	
DOT (n = 2,423)				<0.001
Yes	704 (77.4)	205 (22.6)	909 (37.5)	
No (self-administration)	923 (61.0)	591 (39.0)	1,514 (62.5)	

Data described in absolute (n) and relative (%) frequencies. The total number of cases evaluated was 3,022, and for each variable analysed, cases labelled as ‘blank’ and ‘ignored’ were excluded. Significant values for *P* < 0.05 (χ² test).

eAPP = prison primary care team; DOT = directly observed treatment.

In unadjusted analysis of the socio-economic and clinical dimension, the female sex, age groups of 30–39 years and 40–49 years, and people living with HIV/AIDS were associated with unfavourable outcome of TB. After adjustment, the variable HIV/AIDS remained significant (OR = 0.63; CI 95% 0.44–0.89) (Table 3).

**Table 3.** Analysis of factors associated with unfavourable TB treatment outcomes in population deprived of liberty in Rio Grande do Sul, 2018–2020.

Evaluated dimensions	Bivariate regression		Multivariate regression	
	OR (95% CI)	*P* value	OR (95%)	*P* value
Socio-economic and clinical dimension				
Sex				
Male	1		1	
Female	1.77 (1.18–2.64)	0.006	1.61 (0.86–3.03)	1.134
Age				
18–29 years	1		1	
30–39 years	1.29 (1.09–1.53)	0.003	0.89 (0.67–1.18)	0.425
40–49 years	1.40 (1.13–1.74)	0.002	0.83 (0.57–1.19)	0.302
60 years or older	1.28 (0.68–2.41)	0.439	1.43 (0.61–3.34)	0.406
Skin colour				
White	1		1	
Non-white	0.96 (0.81–1.32)	0.630	1.06 (0.82–1.35)	0.656
Level of education				
Up to 8 years of schooling	1		1	
8 years or more	1.15 (0.95–1.41)	0.160	1.20 (0.93–1.54)	0.169
HIV/AIDS				
Yes	1		1	
No	0.49 (0.40–0.61)	<0.001	0.63 (0.44–0.89)	0.009
Use of PAS				
Yes	1		1	
No	0.91 (0.77–1.06)	0.225	0.89 (0.70–1.13)	0.331
Comorbidities				
Yes	1		1	
No	0.80 (0.63–1.01)	0.055	0.88 (0.64–1.22)	0.452
Government strategies dimension				
Notification by the eAPP				
No	1		1	
Yes	0.59 (0.51–0.69)	<0.001	0.77 (0.60–0.98)	0.036
Entry type				
Transfers	1		1	
Re-entry after abandonment	0.81 (0.58–1.13)	0.215	0.48 (0.28–0.83)	0.008
Relapse	0.57 (0.42–0.78)	<0.001	0.37 (0.22–0.61)	<0.001
New case	0.48 (0.37–0.63)	<0.001	0.37 (0.24–0.57)	<0.001
Diagnostic criteria				0.009
Clinical epidemiology	1		1	
X-ray and histopathology	0.67 (0.44–1.03)	0.066	0.57 (0.26–1.17)	0.124
Bacteriological	0.54 (0.37–0.79)	0.002	0.44 (0.22–0.87)	0.018
DOT				
No	1		1	
Yes	0.45 (0.37–0.55)	<0.001	0.52 (0.40–0.70)	<0.001

Bivariate and multivariate regression considering the outcome of TB cure (cure vs. unfavourable TB treatment outcomes). significant values for *P* < 0.05.

OR = odds ratio; 95% CI = 95% confidence interval; eAPP = prison primary care team; DOT = directly observed treatment.

In unadjusted analysis of government strategies, notifications made by teams external to the prison system, entries due to transfers, clinical epidemiological diagnosis, and self-administered treatment were associated with unfavourable outcome of TB. In the multivariate analysis, the same variables remained associated with unfavourable outcome of TB (Table 3).

## DISCUSSION

TB is an avoidable consequence of incarceration and can be controlled through public policies with targeted strategies^8,9^ and improving coordination between intra- and extra-mural TB control programmes. Identifying TB cases in PDL and understanding the factors that influence this context, the dynamics of disease progression, and, particularly, the factors leading to an unfavourable outcome of treatment are crucial for defining public policies and strategies for disease control.^10,11^ Curing TB means preventing the worsening and death caused by the disease, avoiding transmission, and interrupting the chain of contagion.^12,13^ In this study, government strategies for TB control proved more effective in terms of outcomes compared with the socio-economic and clinical dimension. Planned strategies and actions, such as care provided by the eAPP, based on evidence such as DOT^14^ and clinical and epidemiological protocols implemented across all penal institutions, could be highly effective in disease control.

In Rio Grande do Sul state, eAPP were responsible for a 52.9% increase in TB notifications in PDL between 2014 and 2020.^15^ Analyses indicate greater chances of unfavourable outcome of treatment when notifications are made by other health teams, supporting a study conducted in Belém, Pará state, in which difficulties and deficiencies in TB care within the prison system were attributed to the low coverage of eAPP.^16^ Considering the National Comprehensive Health Care Policy for the Population Deprived of Liberty in the Prison System (Portuguese acronym: PNAISP, *Política Nacional De Atenção Integral à Saúde da População Privada de Liberdade no Sistema Prisional*), the eAPP should engage in actions related to promotion, prevention, assistance, and health surveillance, ensuring comprehensiveness. They are also crucial in coordinating strategic and cross-cutting actions with other institutional sectors to facilitate access to health services within the Health Care Network (Portuguese acronym: RAS, *Redes de Atenção à Saúde*).^17^ It is strongly recommended that municipalities, particularly those with a high TB burden, adhere to the PNAISP and implement eAPP in PDL. The eAPP can be valuable partners of the epidemiological surveillance sector, provided they are trained and empowered to perform diagnosis, notification, follow-up, and outcomes of TB cases. Furthermore, transferring the governance of health systems within prisons from the Ministry of Justice to local governments has yielded promising results in various countries.^18^ The control of TB in prisons should be a political priority to improve the epidemiological situation of TB in general population.

This study also highlighted the importance of treatment adherence and the connection between health services and users. Transfers of the PDL during TB treatment are associated with treatment abandonment^9^ and exert negative impact on disease cure. Between 2014 and 2018, transfers accounted for 16.5% of the notified cases in the PDL in Rio Grande do Sul, with a cure rate of 46.6%.^20^ The ongoing dialogue between health and security sectors in the state has significantly reduced the percentage of transfers, which reached 8.6% in this evaluation.

Regarding diagnosis, its early performance is essential, as half of individuals with the disease may be asymptomatic. In Brazil, the rapid molecular test using Xpert MTB/RIF is recommended as the first choice for PDL, followed by bacteriological confirmation of the case with culture and sensitivity testing.^21^ Sputum smear microscopy is not the test of choice for PDL in places where the rapid molecular test for TB is available since smear microscopy has lower sensitivity compared with the rapid molecular test and does not identify drug resistance.^22^ Strategies that expand early detection are essential to mitigate these impacts. A promising example is sputum pooling, which has proven to be an efficient and low-cost alternative for active screening of TB in PDL, allowing faster diagnosis and reducing unfavourable outcomes.^23^

Our results support the association between DOT and TB cure^24–26^ so that DOT should be the rule. However, the implementation of DOT in the prison system is hindered by overcrowding, insufficient staff, and security concerns, which, in this context, take precedence over health issues.^27,28^

In relation to the socio-economic and clinical dimension, individuals living with HIV/AIDS in the prison system have a 37% higher likelihood of unfavourable outcome. In the prison system, the risk of *Mycobacterium tuberculosis* infection in people living with HIV is three times higher than in the general HIV-positive population.^4^ Several studies have shown an association between TB/HIV co-infection and unfavourable outcomes.^29–32^ The assessment of HIV serostatus represents an opportunity for intervention and a key element in TB control programmes.^4,12^ Therefore, although considered a clinical dimension in this study, HIV/AIDS is also subject to intervention through public policies, strategies, and targeted actions.

Although was not statistically significant, the 47% consumption of PAS is high and may influence adherence to treatment. The types of PAS used were not identified, but in Brazil, smoked and inhaled drugs prevail. Also, the socio-economic and clinical dimension was of lesser importance compared with government strategies, and it is essential to provide concomitant treatment for other diseases and conditions. These factors significantly affect the physical, psychosocial well-being and quality of life of the TB population, which is unfavourable to the healing process.^27^

Limitations include the use of secondary data, which did not allow for cross-referencing of information or identification of the individuals diagnosed, nor could it specify whether TB transmission occurred within the prison system or prior to incarceration. It also did not allow access to data on drug-resistant TB. The lack of more detailed reports on individual and environmental characteristics restricts the ability to determine causal relationships for the higher rates observed in the prison context.

## CONCLUSION

This study highlights the importance of public policy dimensions and strategies aimed at curing TB in the PDL of Rio Grande do Sul, and strongly recommends that municipalities with PDL adhere to the PNAISP, implement the eAPP, and maintain Continuing Education in Health initiatives for professionals working within the prison system. Unfavourable outcomes can be minimised by reducing inequities, aligning with the 2030 Agenda and Sustainable Development Goal 3, which aims to ensure healthy lives and promote well-being for everybody at all ages. To achieve this, it is essential to reduce premature deaths from communicable diseases and improve access to health systems for disease prevention, health promotion, and social protection. The findings of this study provide guidance for TB control strategies within the prison system, emphasising case notification and follow-up by eAPPs, and individuals in prison without established links to health teams, improved diagnostic capabilities, increased proportion of cases undergoing DOT, and care related to the prevention and treatment of HIV/AIDS, particularly TB/HIV co-infection.
